# Uveal melanoma and marital status: a relationship that affects survival

**DOI:** 10.1007/s10792-022-02406-2

**Published:** 2022-07-11

**Authors:** Ahmad Alfaar, Anas Saad, Piotr Chlad, Omneya Ezzat Elsherif, Mohammad Elshami, Catharina Busch, Matus Rehak

**Affiliations:** 1 Experimental Ophthalmology, Campus Virchow-Klinikum, Charite Universitätsmedizin Berlin, Berlin, Germany; 2 Department of Ophthalmology, University Hospital Leipzig, University of Leipzig, Leipzig, Germany; 3 Cleveland Clinic Foundation, Cleveland, OH USA; 4 Family Medicine, Egyptian Fellowship Board, Cairo, Egypt; 5 Family Medicine, Cairo University Hospitals, Cairo, Egypt; 6 Shezlong Telepsychiatry Services, Cairo, Egypt; 7 Department of Ophthalmology, University Hospital of Giessen and Marburg, Giessen, Germany

**Keywords:** Uveal melanoma, Eye cancer, Epidemiology, Psycho-oncology, Marriage, Social status

## Abstract

**Background:**

Marital status influences the presentation and outcome of various cancers. We explored the relationship between marital status and survival of uveal melanoma (UM) and factors influencing this relationship.

**Methods:**

We conducted a retrospective cohort study on patients diagnosed with UM and registered in the Surveillance Epidemiology and End Results program between 1973 and 2017. Cox regression model was conducted to calculate the hazard ratio of overall and cancer-specific survival rate and delineate the effect of each confounder.

**Results:**

The study involved 10,557 patients with a male-to-female ratio of 1:1.1. Most of the diagnosed patients were aged between 40 and 79 years (81%). Married patients (62%) represented the majority, followed by singles (12%), widowed (11%), and then divorced patients (7%). Single patients were the youngest group (mean age of 59.3 years) while widowed patients were the oldest (mean age of 75.8 years). In the Cox regression model for overall survival, married and single patients exhibited the best overall survival (no significant difference in between them), both surpassing divorced and widowed patients. Married patients were at a significantly lower risk to die from UM than divorced patients. Female patients and younger age groups showed the best overall and cancer-specific survival.

**Conclusion:**

Maintained marriages improved the survival of UM patients. Widowed and divorced patients should be included in specially designed support programs during their cancer management.

**Supplementary Information:**

The online version contains supplementary material available at 10.1007/s10792-022-02406-2.

## Introduction

Uveal melanoma is an aggressive tumor of the choroid and ciliary body. It is the most common primary malignant intraocular tumor of the eye in adults. The mean age-adjusted incidence of uveal melanoma in the USA is 5.2 per million. Most cases (97.8%) occur in the white population, similar to that reported from European countries [1]. Studies have shown that it has different developmental mechanisms than melanoma of the skin [2].

Cancer survival depends on modifiable and non-modifiable factors. Mental health is one of the factors that influence the patient’s perception of and adherence to treatment plans and follow-up visits. Part of the mental health practices is social status, which includes marriage. In some studies, marriage appears to have a protective effect and contributes to a better quality of life, which may lead to improved outcomes [3]. Besides the psychological support, it is likely that spousal resources also contribute to differentials in survival due to their own network of resources. Multiple studies have shown that marriage is associated with early diagnosis and better survival of various tumors, including skin tumors [3–5].

This study investigates the relationship between marital status and its effect on diagnosis, disease-specific, and overall survival of uveal melanoma patients.

## Methods

### Study design and population

The study is a retrospective cohort study of patients registered in 18 US cancer registries. We have extracted the data from the Surveillance, Epidemiology, and End Results (SEER) program of the US national cancer institute between 1973 and 2017. As a reported disease, the data were collected by SEER and anonymously distributed. Therefore, it is considered nonhuman subject data and waived from IRB approval. Our study adhered to the ethical principles of the Declaration of Helsinki and its amendments. The data were extracted using SEER*Stat Program 8.3.8, case listing session for the incidence database “SEER 18 Regs Research Data + Hurricane Katrina Impacted Louisiana Cases, Nov 2018 Sub (1975–2016 varying)—Linked To County Attributes—Total U.S., 1969–2017 Counties.” The patients were selected using melanoma as a histology group for WHO 2008 classification and C69.3-choroid and C69.4-ciliary body and iris as primary sites using ICD-O-3 topography classification. Only patients with malignant tumors and known age were included. SEER historical classification was used for classifying the patients in local and metastatic tumors.

## Statistical analysis

The continuous data were presented as mean (with 95% confidence interval) while discrete data were presented as number (N%). Distributions were shown on histograms, and a comparison of means was conducted according to the normality of the continuous data. *Z*-tests were used to compare column proportions in the characteristics tables. Bonferroni test was used to adjust the p-values for multiple comparisons in the characteristics table. A *p*-value of < 0.05 was considered significant. The time-to-event/survival analysis was conducted using the Kaplan–Meier method, and comparisons between subgroups were made using the log-rank test. Besides Kaplan–Meier estimate, life tables and Cox regression were conducted to study the survival/time-to-event analysis. Cox regression analysis was conducted to adjust for the effect of age, sex, stage, site of the tumor (choroid vs. ciliary body/iris), and rural/urban living factors described in Fig. 3. Due to the observed variations in age groups and sex and their expected effect, we have conducted two Cox regression model analyses to adjust for the variations in age, sex, stage, tumor site, and factors related to the availability of services like the rural/urban lifestyle.

For a subset of patients whose tumor size was available (*N* = 1795 for overall survival and *N* = 1777 for cancer-specific survival), we conducted a separate Cox regression analysis considering measured tumor basal diameter and thickness (Depth). The reference group in COX models was the divorced patients. The analysis of individual patients was conducted using IBM SPSS version 26.

## Results

### Patients’ characteristics

The study included 10,557 patients diagnosed with uveal melanoma between 1973 and 2017 and born between 1884 and 2007. Males were slightly more than females (*n* = 5525, 52.3% vs. *n* = 5032, 47.7%, respectively, the male-to-female ratio was 1:1.1 (Table 1)). Most of the patients were diagnosed in the age group of 40–59 years (*n* = 3660, 34.7%) and 60–79 years (*n* = 4883, 46.3%). White ethnicity represented most of the sample (*n* = 10,231, 96.9%), mainly non-Hispanic (*n* = 9782, 92.7%). Choroid was the main affected uveal part (*n* = 9032, 85.6%) compared to ciliary body (*n* = 1525, 14.4%).

**Table 1:** Patients’ Characteristics

Characteristics*		Single (never married)		Married (including common law)		Widowed		Divorced		Separated		Unmarried or Domestic Partner		Total	
		*N* = 1292		*N* = 6518		*N* = 1169		*N* = 719		*N* = 84		*N* = 7		*N* = 9789	
		N	Col N %s	N	Col N %	N	Col N %	N	Col N %	N	Col N %	N	Col N %	N	Col N %
*Marital status at diagnosis*															
Sex	Female	593	45.9%	2675	41.0%	923	79.0%	425	59.1%	51	60.7%	1	14.3%	4668	47.7%
	Male	699	54.1%	3843	59.0%	246	21.0%	294	40.9%	33	39.3%	6	85.7%	5121	52.3%
Age Group	0–19	59	4.6%	0	0.0%	0	0.0%	0	0.0%	0	0.0%	0	0.0%	59	0.6%
	20–39	230	17.8%	458	7.0%	3	0.3%	32	4.5%	10	11.9%	0	0.0%	733	7.5%
	40–59	506	39.2%	2484	38.1%	70	6.0%	320	44.5%	29	34.5%	3	42.9%	3412	34.9%
	60–79	414	32.0%	3080	47.3%	641	54.8%	337	46.9%	38	45.2%	3	42.9%	4513	46.1%
	80 +	83	6.4%	496	7.6%	455	38.9%	30	4.2%	7	8.3%	1	14.3%	1072	11.0%
Race	White	1240	97.3%	6339	98.2%	1148	98.4%	696	98.0%	82	97.6%	6	100.0%	9511	98.1%
	Black	16	1.3%	38	0.6%	7	0.6%	7	1.0%	1	1.2%	0	0.0%	69	0.7%
	American Indian/Alaska Native	2	0.2%	17	0.3%	2	0.2%	3	0.4%	0	0.0%	0	0.0%	24	0.2%
	Asian or Pacific Islander	16	1.3%	58	0.9%	10	0.9%	4	0.6%	1	1.2%	0	0.0%	89	0.9%
Rural–Urban Continuum 2003	Rural	15	1.2%	139	2.1%	27	2.3%	11	1.5%	0	0.0%	0	0.0%	192	2.0%
	Metropolitan	1158	89.8%	5528	85.1%	992	85.0%	625	87.5%	76	90.5%	7	100.0%	8386	85.9%
	Urban	117	9.1%	832	12.8%	148	12.7%	78	10.9%	8	9.5%	0	0.0%	1183	12.1%
Laterality	Right	639	49.5%	3260	50.3%	593	51.3%	339	47.3%	37	44.6%	4	57.1%	4872	50.1%
	Left	650	50.4%	3218	49.7%	562	48.7%	378	52.7%	46	55.4%	3	42.9%	4857	49.9%
	Bilateral	1	0.1%	0	0.0%	0	0.0%	0	0.0S%	0	0.0%	0	0.0%	1	0.0%
Primary Site	Choroid	1082	83.7%	5601	85.9%	1000	85.5%	633	88.0%	66	78.6%	6	85.7%	8388	85.7%
	Ciliary body / Iris	210	16.3%	917	14.1%	169	14.5%	86	12.0%	18	21.4%	1	14.3%	1401	14.3%
SEER stage (1973–2015)	Localized	1016	91.4%	5118	91.6%	897	89.6%	559	90.6%	67	91.8%	5	100.0%	7662	91.3%
	Regional	76	6.8%	378	6.8%	76	7.6%	44	7.1%	2	2.7%	0	0.0%	576	6.9%
	Distant	20	1.8%	89	1.6%	28	2.8%	14	2.3%	4	5.5%	0	0.0%	155	1.8%
Derived AJCC Stage Group, 6th ed (2004–2015)	I	238	37.4%	1068	37.5%	146	33.3%	132	37.4%	14	42.4%	1	25.0%	1599	37.1%
	II	246	38.7%	1234	43.4%	191	43.5%	144	40.8%	11	33.3%	0	0.0%	1826	42.4%
	III	140	22.0%	494	17.4%	88	20.0%	65	18.4%	7	21.2%	3	75.0%	797	18.5%
	IV	12	1.9%	50	1.8%	14	3.2%	12	3.4%	1	3.0%	0	0.0%	89	2.1%
Grade	Well differentiated; Grade I	21	41.2%	64	35.6%	7	18.4%	5	26.3%	0	0.0%	0	0.0%	97	33.4%
	Moderately differentiated; Grade II	21	41.2%	80	44.4%	23	60.5%	9	47.4%	2	100.0%	0	0.0%	135	46.6%
	Poorly differentiated; Grade III	8	15.7%	32	17.8%	6	15.8%	5	26.3%	0	0.0%	0	0.0%	51	17.6%
	Undifferentiated; anaplastic; Grade IV	1	2.0%	4	2.2%	2	5.3%	0	0.0%	0	0.0%	0	0.0%	7	2.4%

*only cases with known status were included

**Fig. 1:**
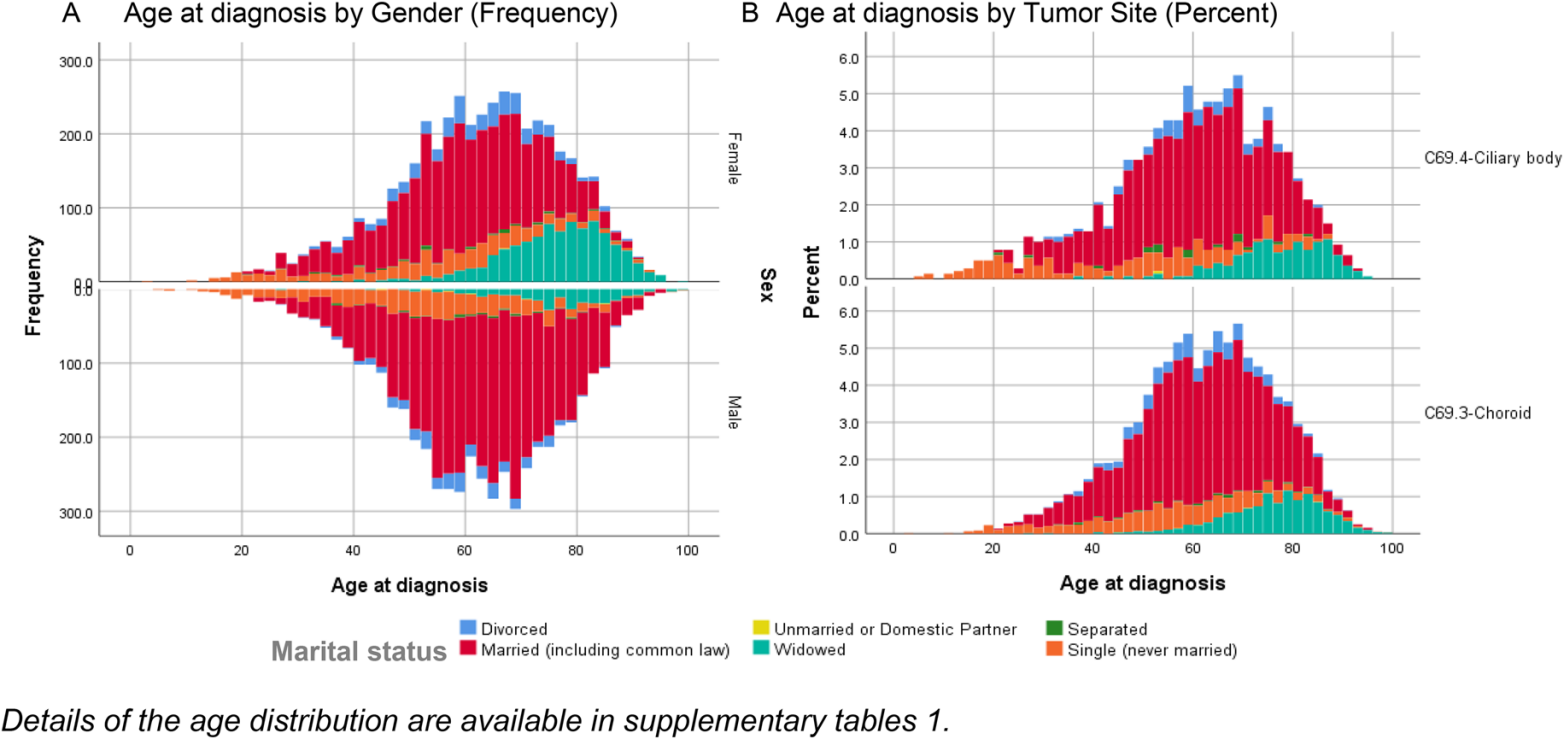
Distribution of marital status for each age group in each gender **A** and tumor location **B**

The mean age at diagnosis was 61.5 years (CI 95%: 61.2–61.8). Females were slightly older than males (62.0 years [95% CI 61.6–62.4] vs. 61.0 years [95%CI 60.6–61.4 years]; Supplementary Table 1). Divorced women were significantly older than divorced men (60.8 years [95%CI 59.7–62.0] vs. 58.8 years [95%CI 57.5–60.0], *p* = 0.02), while married men were significantly older than married women (61.9 years [CI 95%: 61.4–62.3] vs. 59.2 years [CI 95%: 58.7–59.7], *p* < 0.001). Widowed women were more predominant than widowed men and older than other groups (Fig. 1). Most of the patients (*n* = 8386, 85.9%) were living in metropolitan regions.

### Marriage

The sample included 6518 (61.7%) married patients, 1292 single (12.2%), 1169 (11.1%) widowed, 719 (6.8%) divorced, and 84 (0.9%) separated patients. Seven patients were either unmarried or with a domestic partner and 768 (7.3%) patients with unknown status. Single patients represented the youngest group with a mean age of 53.2 years (95%CI: 52.2–54.2). Widowed patients represented the most senior group with a mean age of 75.8 years (95%CI 75.2–76.4) (Supplementary Table 1). The age and gender showed a significant difference among the studied groups (ANOVA *p* < 0.001).

### Survival

**Fig. 2:**
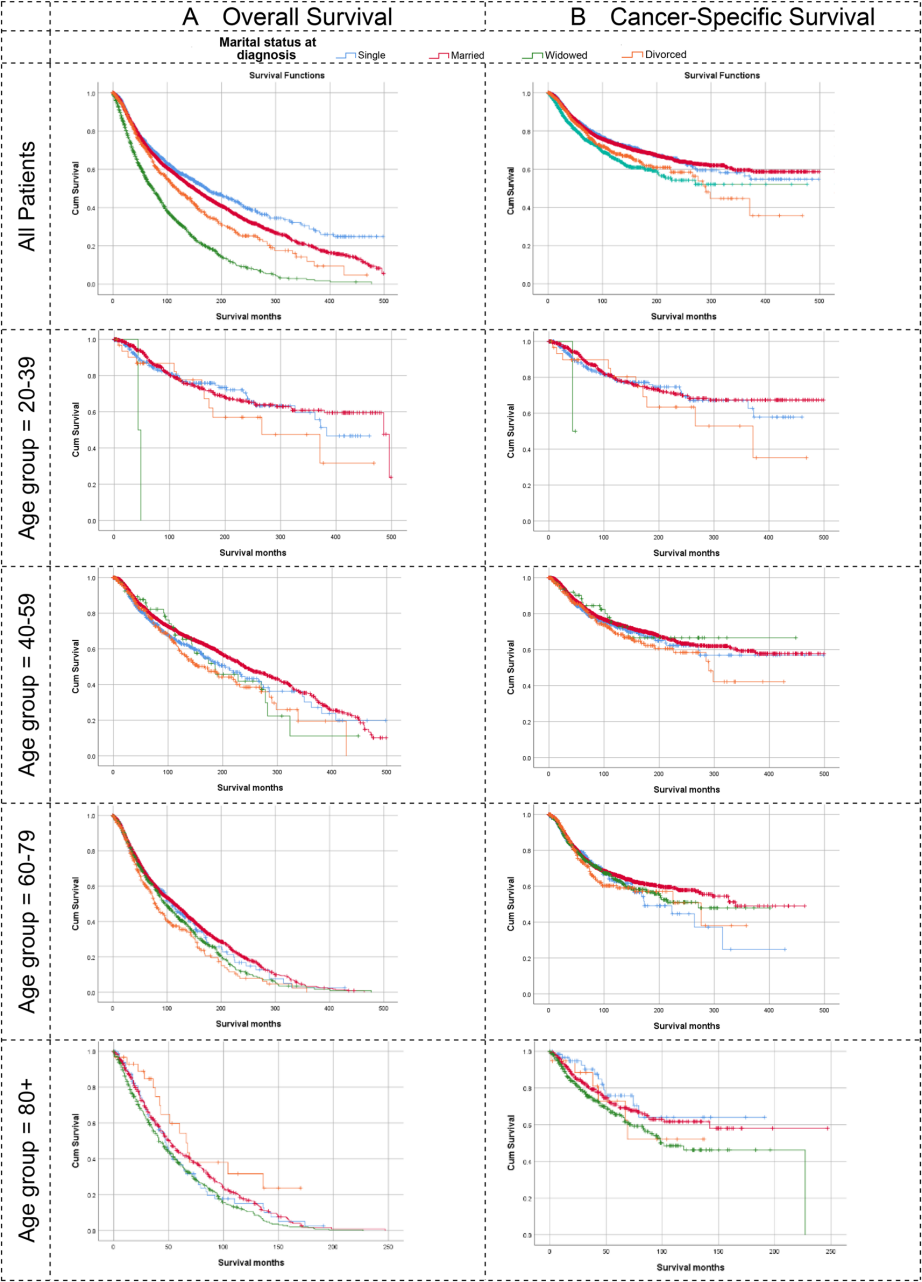
Survival by Marriage and Age Group at Diagnosis. **A**. Overall Survival, **B**. Cancer-Specific Survival. Details of the analysis are available in supplementary tables 2–5

The overall crude survival at 10 years after diagnosis showed a survival descent from singles (50%) to married (45%) to divorced (36%) followed by widowed patients (19%) (Supplementary Tables 2 & 3, Fig. 2). The pairwise Wilcoxon test showed a significant difference between widowed (*p* < 0.001), and divorced (*p* < 0.02), and between them and the other two groups (Supplementary Table 3). The 10-year cancer-specific survival showed a similar pattern with less variation between the groups and an almost identical survival probability between single (70%) and married patients (69%), both higher than divorced (63%) and widowed patients (60%) (Supplementary Tables 4 & 5, Fig. 2).

**Fig. 3:**
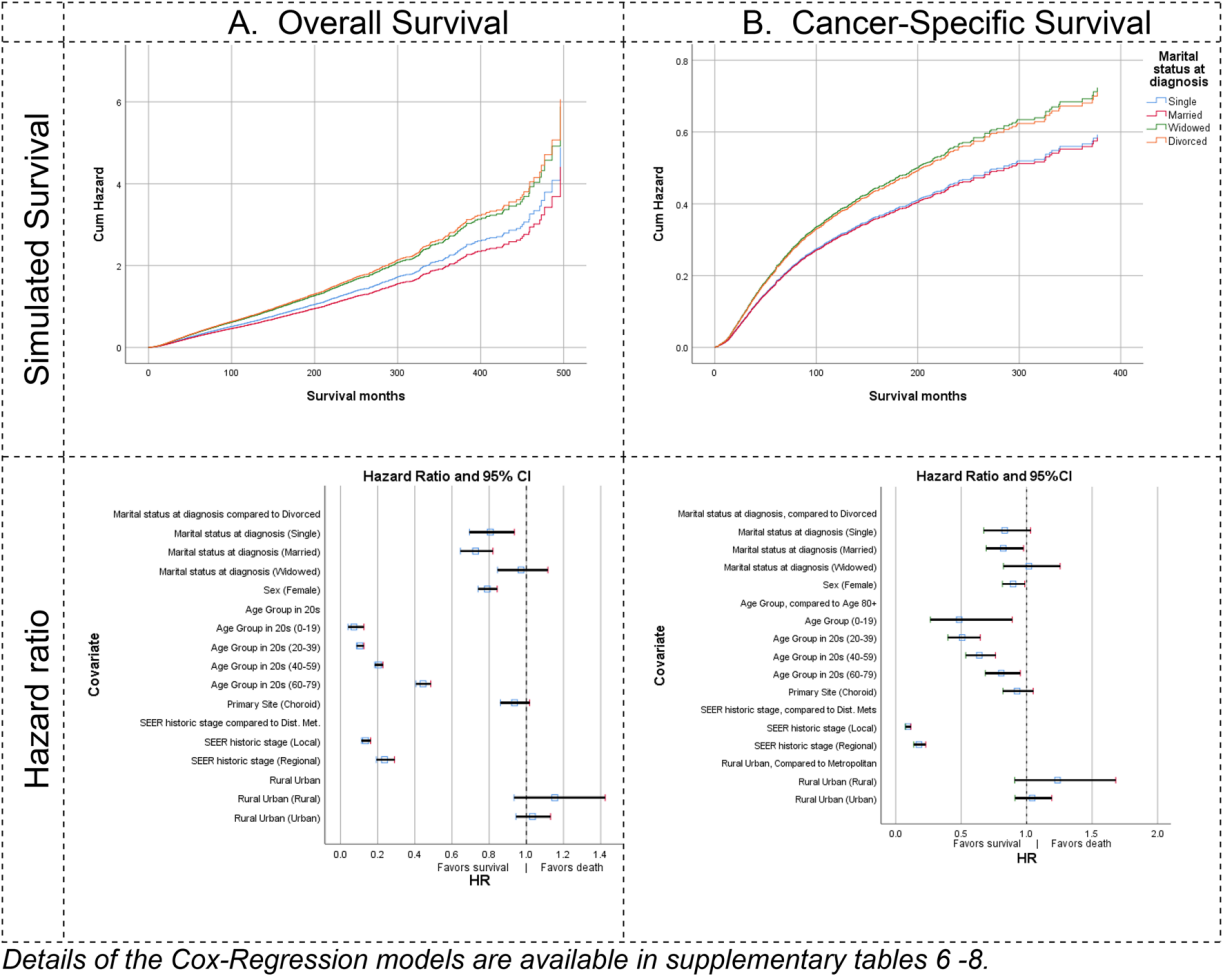
Hazard Ratio of Overall **A** and Cancer-Specific Survival **B**. Please note the different in y-axis scale

In the first Cox regression model, married patients showed the most favorable overall survival risk (HR = 0.73, 95%CI 0.65–0.82) followed by single patients (HR = 0.81, 95%CI 0.69—0.94) with no significant difference between both. In contrast, widowed patients showed lower favorable risk (HR = 0.97, 95%CI 0.85–1.12) but not significantly different from divorced (Fig. 3, Supplementary Table 6). Women, patients with local tumors and younger age groups, showed more favorable risk than other groups.

Married patients showed significant lower risk (HR = 0.82, 95%CI 0.69–0.98) than divorced to die from uveal melanoma. Women (HR = 0.90, 95%CI 0.82–0.99) and younger older age groups also showed better cancer-specific survival (Fig. 3, Supplementary Table 7). COX regression models that considered tumor dimensions significantly influenced overall and cancer-specific survival (Supplementary Table 8).

Both overall and cancer-specific survival models presented that married patients have had a lower hazard ratio than single and widowed patients (Supplementary Table 8). However, these differences were not statistically significant. Women, patients with choroidal tumors (*p* < 0.01), local tumors (*p* < 0.001), and smaller basal tumor diameter (*p* < 0.01) showed significantly favorable survival in overall and cancer-specific models.

## Discussion

Uveal melanoma is an aggressive tumor of the uveal tract characterized by early metastasis and general poor survival. It is of high interest to identify factors that impact survival rates. We showed that overall and cancer-specific survival differed among patients with different marital statuses. We found that unmarried, widowed, or divorced patients are at a significantly higher risk of death than married patients. Those results were observed in all analyzed age groups. Previous studies that included the most common cancers, like pulmonary, colorectal or pancreatic cancer, already showed a higher risk of metastatic disease and death from cancer in non-married compared to married patients [3–5].

Marital status might impact the stage of diagnosis as the spouses can encourage to seek medical attention after experiencing disturbing eye symptoms. Spouses may also support the patients in the decision of possibly sight-threatening or even definitive therapy. It could be presumed that married patients present a better adherence to postoperative screening procedures similar to the treatment adherence among married patients with systemic disorders [6, 7]. Impaired adherence may delay the diagnosis of metastases and the beginning of the proper therapy. Previous studies showed that married cancer patients’ survival is affected by their spouses’ educational attainments, net of education, income and discrepancies in spouses’ ages, education, and incomes.

One study highlighted that after adjusting for known confounders, unmarried patients were at significantly higher risk of presentation with metastatic cancer, undertreatment, and death resulting from their cancer. It highlighted the impact of social support on cancer detection, treatment, and survival [3]. The survival benefit associated with marriage was larger than the published survival benefit of chemotherapy [3]. Moreover, unmarried patients had a higher risk of being diagnosed at a late stage among men and women, the magnitude of the effect varied by sex. Among married, single, and divorced or separated patients, men had more than a 50% increase in the risk of late-stage diagnosis when compared with women. However, widowed men and widowed women were not statistically different in their stage at diagnosis [8], which emphasizes the vital role of marital status. A previous study by Damato et al. has shown better patients’ reported outcomes by married patients [9].

One important reason for better survival among married patients could be the psychological support by the spouses. Married patients can experience less anxiety and depression after diagnosing a tumor, as they can share the burden with a partner [10]. Depression may be a significant factor impacting mortality as it affects the treatment participation [11, 12] or the readiness to undergo a definitive treatment [13]. Having good psychological support enhances the mental health of survivors by many means. First, the psychological state is related to the hypothalamic–pituitary–adrenal axis (HPA axis) activity and the cortisol level in the blood which changes while suffering from depression and anxiety [14, 15]. These changes affect the immunity cells and their mediators in the plasma and increase the vulnerability to more side effects of medications in patients suffering from mental health issues [16]. Secondly, the response to treatments provided to patients is better in patients with healthy psychological status and support. Third, patients with mental health problems have compliance difficulties either on medications or on follow-up visits, which is essential to detect any further problems to be dealt with as fast as possible [17–19].

Although our study is a population-based study with a long duration, which spanned about 34 years, it includes limitations imposed by its retrospective nature. Data on TNM classification, as an essential confounder of survival rate, were not available in a sufficient manner and thus could not be included in the analysis. Other possible confounders on the survival rate, including those related to the patients themselves, available psychological support, treatment, treating facility, and possible financial and educational privilege, were also not available. A further limitation of our study is a limited sensitivity to changes in patients’ social status due to the registry-based nature.

Our study shows that marital status can have an impact on tumor survival in patients with uveal melanoma. We identified unmarried, widowed, or divorced patients have the lowest survival rate. An early and constant social and psychological support of these vulnerable patients might improve therapy outcomes and survival rates.

## Supplementary Information

Below is the link to the electronic supplementary material.

Supplementary file1 (DOCX 337 KB)

Supplementary file2 (XLSX 38 KB)

## Data Availability

Not applicable.
