# Supplementary material for: Uveal melanoma and marital status: a relationship that affects survival
Source: Int Ophthalmol. 2022 Jul 11;42(12):3857–67. doi: 10.1007/s10792-022-02406-2 (PMC9617958; doi:10.1007/s10792-022-02406-2)
Supplement: Supplementary file 1 — Supplementary file1 (DOCX 337 KB) [file 10792_2022_2406_MOESM1_ESM.docx]

Supplementary Data

Supplementary Figure 1. Cancer Specific Survival, and related Hazard Function

Supplementary table 1: Age and survival months in the groups

Supplementary table 2: Overall Survival Details, Log-Rank Test

Supplementary table 3: Life Tables analysis for Overall Survival

Supplementary table 4: Cancer-specific Survival Details, Log-Rank Test

Supplementary table 5: Life Tables analysis for Cancer-Specific Survival

Supplementary table 6: Details of Cox-Regression Model for overall survival.

Supplementary table 7: Details of Cox-Regression Model for Cancer-Specific survival.

Supplementary table 8: Details of Cox-Regression Model for Overall and Cancer-Specific survival with considering tumor size.

Supplementary Figure 1. Cancer Specific Survival- related Hazard Function


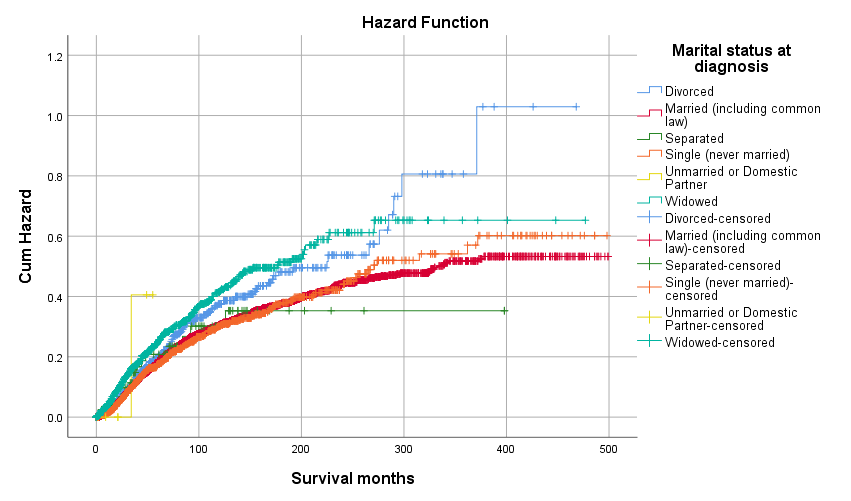


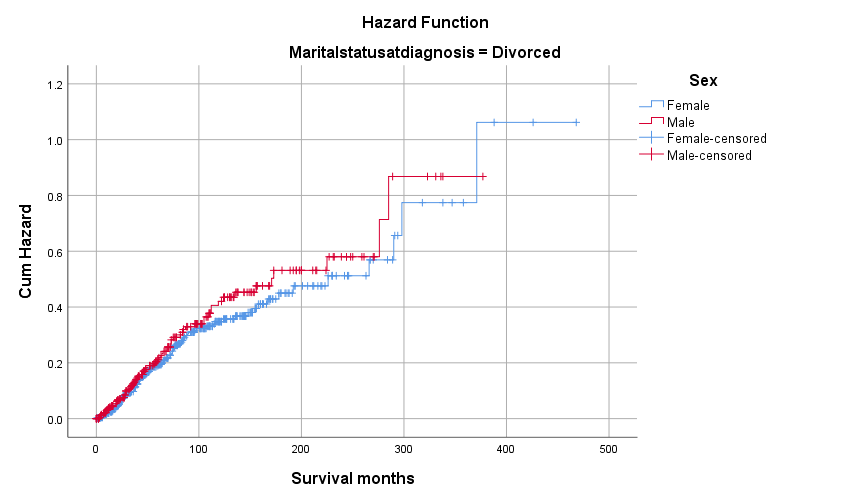

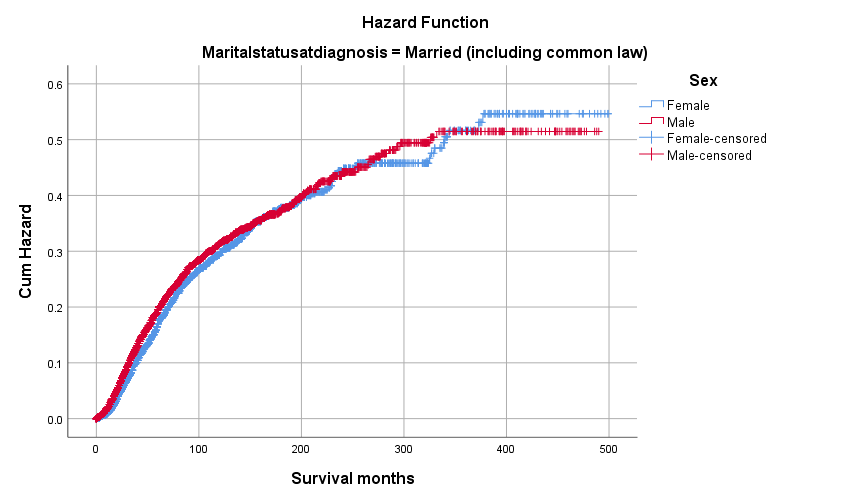


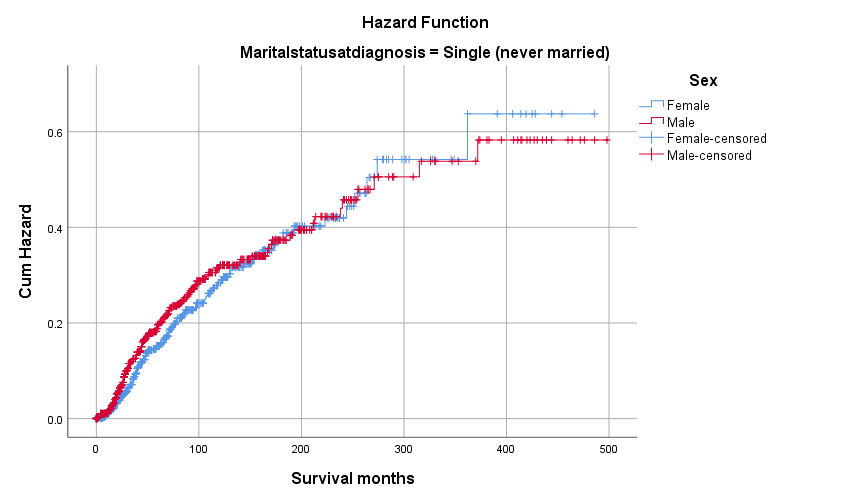

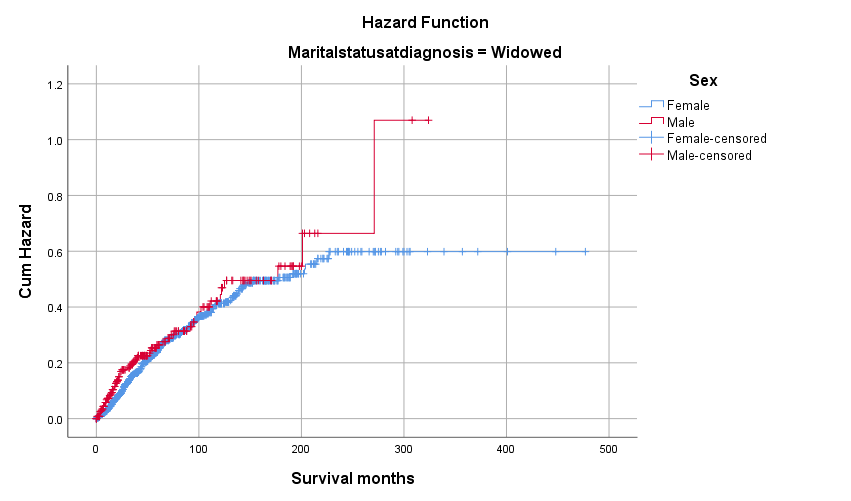


**Urban – Metropolitan**


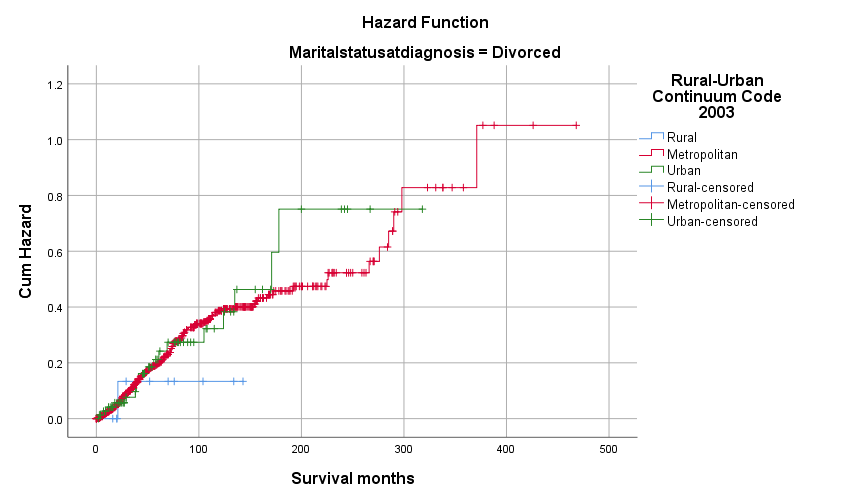

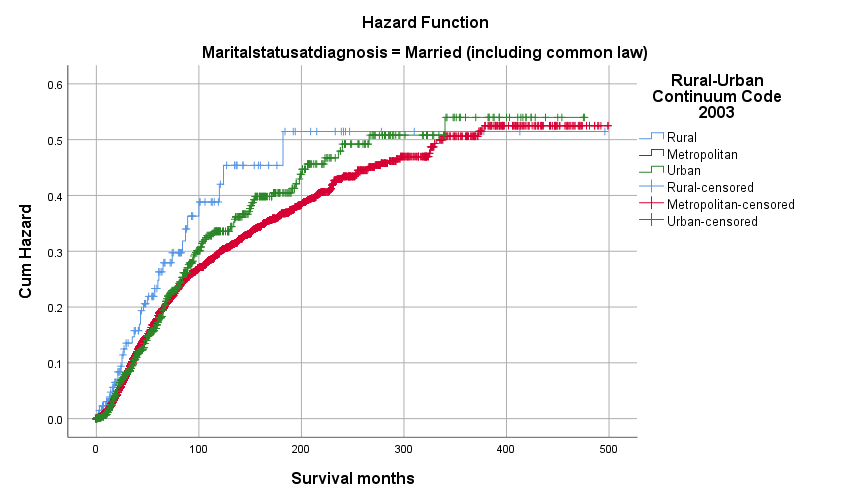


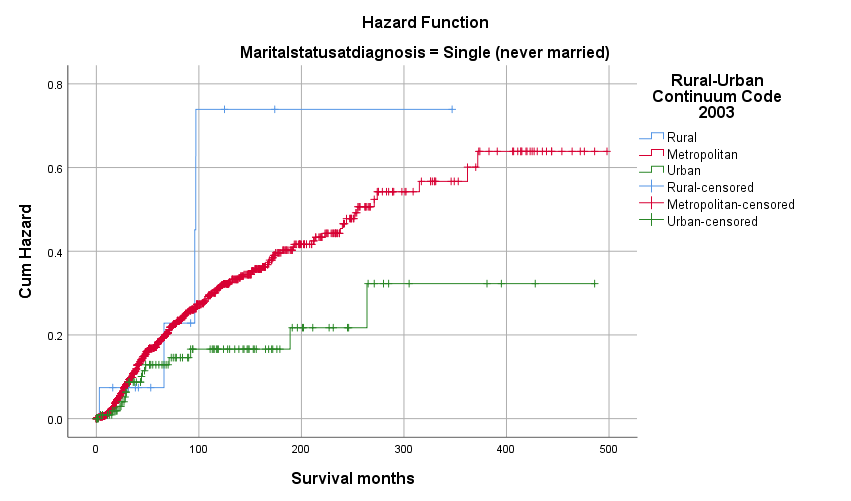

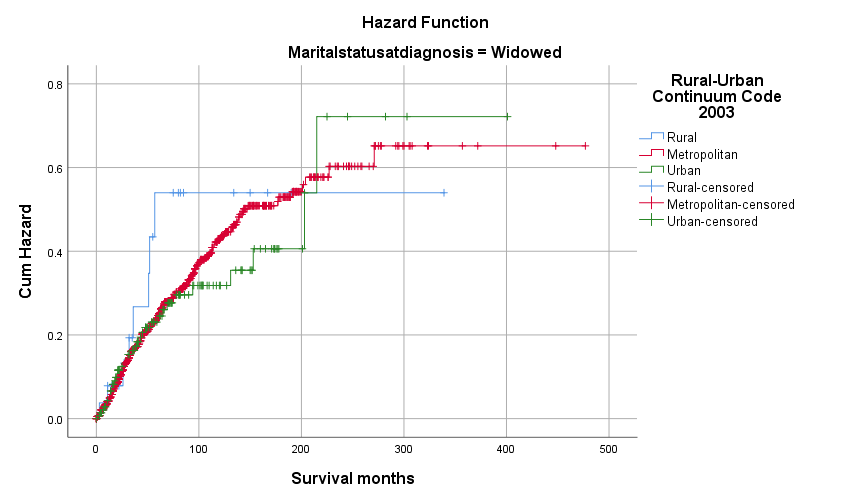


Supplementary Table 1: Age at diagnosis and survival time of the patients

|  |  |  | Sex | | | | | | | | | | | | | |
| --- | --- | --- | --- | --- | --- | --- | --- | --- | --- | --- | --- | --- | --- | --- | --- | --- |
|  |  |  | Female | | | | | Male | | | | Total | | | | |
|  |  |  | Mean | 95.0% Lower CL | 95.0% Upper CL | SD | Mean | | 95.0% Lower CL | 95.0% Upper CL | SD | Mean | 95.0% Lower CL | 95.0% Upper CL | SD |  |
| Age at diagnosis | Marital status at diagnosis | Divorced ^*1^ | 60.8 | 59.7 | 62.0 | 12.1 | 58.8 | | 57.5 | 60.0 | 10.9 | 60.0 | 59.1 | 60.8 | 11.6 |  |
|  |  | Married (including common law) ^*2^ | 59.2 | 58.7 | 59.7 | 13.2 | 61.9 | | 61.4 | 62.3 | 13.4 | 60.8 | 60.5 | 61.1 | 13.4 |  |
|  |  | Separated | 58.9 | 54.2 | 63.7 | 16.8 | 59.9 | | 55.5 | 64.3 | 12.4 | 59.3 | 56.0 | 62.6 | 15.2 |  |
|  |  | Single (never married) | 54.2 | 52.7 | 55.7 | 18.4 | 52.3 | | 51.0 | 53.6 | 17.6 | 53.2 | 52.2 | 54.2 | 18.0 |  |
|  |  | Unmarried or Domestic Partner | 66.0 |  |  |  | 61.7 | | 46.5 | 76.8 | 14.4 | 62.3 | 50.0 | 74.6 | 13.3 |  |
|  |  | Widowed | 75.8 | 75.2 | 76.4 | 9.8 | 75.8 | | 74.4 | 77.1 | 10.5 | 75.8 | 75.2 | 76.4 | 9.9 |  |
|  |  | Total | 62.0 | 61.6 | 62.4 | 15.1 | 61.0 | | 60.6 | 61.4 | 14.6 | 61.5 | 61.2 | 61.8 | 14.8 |  |

Significance:

*1 B( .022)

*2 A(< .0005)

Results are based on two-sided tests assuming equal variances. For each significant pair, the key of the smaller category appears in the category with the larger mean.

Significance level for upper case letters (A, B, C): .05

a. This category is not used in comparisons because the sum of case weights is less than two.

b. Tests are adjusted for all pairwise comparisons within a row of each innermost subtable using the Bonferroni correction.

Supplementary table 2: Overall Survival Details, Log-Rank Test

| **Case Processing Summary** | | | | |
| --- | --- | --- | --- | --- |
| Marital status at diagnosis | Total N | N of Events | Censored | |
|  |  |  | N | Percent |
| Divorced | 719 | 349 | 370 | 51.5% |
| Married (including common law) | 6517 | 2944 | 3573 | 54.8% |
| Separated | 84 | 44 | 40 | 47.6% |
| Single (never married) | 1292 | 494 | 798 | 61.8% |
| Unmarried or Domestic Partner | 7 | 3 | 4 | 57.1% |
| Widowed | 1169 | 837 | 332 | 28.4% |
| Overall | 9788 | 4671 | 5117 | 52.3% |

| **Means and Medians for Survival Time** | | | | | | | | |
| --- | --- | --- | --- | --- | --- | --- | --- | --- |
| Marital status at diagnosis | Mean^a^ | | | | Median | | | |
|  | Estimate | Std. Error | 95% Confidence Interval | | Estimate | Std. Error | 95% Confidence Interval | |
|  |  |  | Lower Bound | Upper Bound |  |  | Lower Bound | Upper Bound |
| Divorced | 161.8 | 7.9 | 146.3 | 177.3 | 118.0 | 8.4 | 101.5 | 134.5 |
| Married (including common law) | 196.4 | 3.0 | 190.4 | 202.3 | 150.0 | 3.7 | 142.8 | 157.2 |
| Separated | 148.0 | 20.4 | 108.0 | 188.0 | 113.0 | 15.2 | 83.2 | 142.8 |
| Single (never married) | 229.0 | 8.2 | 212.9 | 245.1 | 173.0 | 12.8 | 148.0 | 198.0 |
| Unmarried or Domestic Partner | 41.0 | 6.1 | 29.1 | 52.9 | 49.0 | 11.9 | 25.8 | 72.2 |
| Widowed | 102.3 | 3.4 | 95.6 | 109.0 | 74.0 | 3.7 | 66.7 | 81.3 |
| Overall | 184.0 | 2.4 | 179.3 | 188.7 | 133.0 | 2.7 | 127.8 | 138.2 |
| a. Estimation is limited to the largest survival time if it is censored. | | | | | | | | |

| **Pairwise Comparisons** | | | | | | | | | | | | | | |
| --- | --- | --- | --- | --- | --- | --- | --- | --- | --- | --- | --- | --- | --- | --- |
|  | Marital status at diagnosis | Divorced | | Married (including common law) | | Separated | | Single (never married) | | Unmarried or Domestic Partner | | Widowed | |  |
|  |  | *Χ^2^* | Sig. | *Χ^2^* | Sig. | *Χ^2^* | Sig. | *Χ^2^* | Sig. | *Χ^2^* | Sig. | *Χ^2^* | Sig. |  |
| Log Rank (Mantel-Cox) | Divorced |  |  | 12.1 | .001 | .6 | .448 | 21.1 | .000 | 3.9 | .048 | 67.0 | .000 |  |
|  | Married (including common law) | 12.1 | .001 |  |  | 4.2 | .040 | 8.1 | .004 | 4.7 | .030 | 336.9 | .000 |  |
|  | Separated | .6 | .448 | 4.2 | .040 |  |  | 7.5 | .006 | 2.5 | .111 | 6.4 | .012 |  |
|  | Single (never married) | 21.1 | .000 | 8.1 | .004 | 7.5 | .006 |  |  | 5.1 | .023 | 225.9 | .000 |  |
|  | Unmarried or Domestic Partner | 3.9 | .048 | 4.7 | .030 | 2.5 | .111 | 5.1 | .023 |  |  | .8 | .361 |  |
|  | Widowed | 67.0 | .000 | 336.9 | .000 | 6.4 | .012 | 225.9 | .000 | .8 | .361 |  |  |  |

*Χ^2^ = Chi-Square, Sig. = Significance in p-value*

Supplementary table 3: Life Tables analysis for Overall Survival

| Group/  Controls | Months | N  Entering Interval | N  Withdrawing during Interval | N  Exposed to Risk | N  of Terminal Events | %  Terminating | % Surviving | Cum. % Surviving at EI | SE of CP Surviving at EI | Pairwise Wilcoxon (Gehan) Sig. |
| --- | --- | --- | --- | --- | --- | --- | --- | --- | --- | --- |
| All Patients, MST = 139.24 m | 0 | 10556 | 2420 | 9346.0 | 2603 | 27.9% | 72.1% | 72.1% | 0.00 |  |
|  | 60 | 5533 | 1329 | 4868.5 | 1224 | 25.1% | 74.9% | 54.0% | 0.01 |  |
|  | 120 | 2980 | 931 | 2514.5 | 582 | 23.1% | 76.9% | 41.5% | 0.01 |  |
|  | 180 | 1467 | 448 | 1243.0 | 285 | 22.9% | 77.1% | 32.0% | 0.01 |  |
|  | 240 | 734 | 463 | 502.5 | 271 | 53.9% | 46.1% | 14.7% | 0.01 |  |
| A. Single, MST= 177.1 M | 0 | 1292 | 341 | 1121.5 | 276 | 24.6% | 75.4% | 75.4% | 0.01 | C, D |
|  | 60 | 675 | 186 | 582.0 | 123 | 21.1% | 78.9% | 59.5% | 0.02 |  |
|  | 120 | 366 | 134 | 299.0 | 50 | 16.7% | 83.3% | 49.5% | 0.02 |  |
|  | 180 | 182 | 60 | 152.0 | 21 | 13.8% | 86.2% | 42.7% | 0.02 |  |
|  | 240 | 101 | 77 | 62.5 | 24 | 38.4% | 61.6% | 26.3% | 0.03 |  |
| B. Married, MST=155.1 M | 0 | 6517 | 1484 | 5775.0 | 1505 | 26.1% | 73.9% | 73.9% | 0.01 | C, D |
|  | 60 | 3528 | 855 | 3100.5 | 706 | 22.8% | 77.2% | 57.1% | 0.01 |  |
|  | 120 | 1967 | 630 | 1652.0 | 351 | 21.2% | 78.8% | 45.0% | 0.01 |  |
|  | 180 | 986 | 289 | 841.5 | 180 | 21.4% | 78.6% | 35.4% | 0.01 |  |
|  | 240 | 517 | 315 | 359.5 | 202 | 56.2% | 43.8% | 15.5% | 0.01 |  |
| C. Widowed, MST= 77.8 M | 0 | 1169 | 164 | 1087.0 | 467 | 43.0% | 57.0% | 57.0% | 0.02 | A, B, D |
|  | 60 | 538 | 79 | 498.5 | 207 | 41.5% | 58.5% | 33.4% | 0.02 |  |
|  | 120 | 252 | 53 | 225.5 | 94 | 41.7% | 58.3% | 19.4% | 0.01 |  |
|  | 180 | 105 | 22 | 94.0 | 45 | 47.9% | 52.1% | 10.1% | 0.01 |  |
|  | 240 | 38 | 14 | 31.0 | 24 | 77.4% | 22.6% | 2.3% | 0.01 |  |
| D. Divorced, MST= 119.86 M | 0 | 719 | 174 | 632.0 | 176 | 27.8% | 72.2% | 72.2% | 0.02 | A, B, C |
|  | 60 | 369 | 88 | 325.0 | 100 | 30.8% | 69.2% | 50.0% | 0.02 |  |
|  | 120 | 181 | 58 | 152.0 | 43 | 28.3% | 71.7% | 35.8% | 0.02 |  |
|  | 180 | 80 | 24 | 68.000 | 18 | 26.5% | 73.5% | 26.3% | 0.03 |  |
|  | 240 | 38 | 26 | 25.000 | 12 | 48.0% | 52.0% | 13.7% | 0.03 |  |

N: Number, EI: End of Interval, Cum. %: Cumulative Proportion, %: Proportion, PD: Probability Density, SE: Standard Error, Sig.: Significance (p-value of the test)

Supplementary table 4: Cancer-specific Survival Details, Log-Rank Test

| **Case Processing Summary** | | | | |
| --- | --- | --- | --- | --- |
| Marital status at diagnosis | Total N | N of Events | Censored | |
|  |  |  | N | Percent |
| Divorced | 719 | 173 | 546 | 75.9% |
| Married (including common law) | 6517 | 1390 | 5127 | 78.7% |
| Separated | 84 | 17 | 67 | 79.8% |
| Single (never married) | 1292 | 266 | 1026 | 79.4% |
| Unmarried or Domestic Partner | 7 | 1 | 6 | 85.7% |
| Widowed | 1169 | 295 | 874 | 74.8% |
| Overall | 9788 | 2142 | 7646 | 78.1% |

| **Means and Medians for Survival Time** | | | | | | | | |
| --- | --- | --- | --- | --- | --- | --- | --- | --- |
| Marital status at diagnosis | Mean^a^ | | | | Median | | | |
|  | Estimate | Std. Error | 95% Confidence Interval | | Estimate | Std. Error | 95% Confidence Interval | |
|  |  |  | Lower Bound | Upper Bound |  |  | Lower Bound | Upper Bound |
| Divorced | 274.6 | 15.0 | 245.2 | 304.0 | 290.0 | 15.0 | 260.7 | 319.3 |
| Married (including common law) | 340.8 | 4.0 | 332.8 | 348.7 | . | . | . | . |
| Separated | 295.7 | 22.0 | 252.5 | 338.8 | . | . | . | . |
| Single (never married) | 333.3 | 9.6 | 314.4 | 352.2 | . | . | . | . |
| Unmarried or Domestic Partner | 48.0 | 5.7 | 36.8 | 59.2 | . | . | . | . |
| Widowed | 291.5 | 10.6 | 270.7 | 312.4 | . | . | . | . |
| Overall | 332.4 | 3.5 | 325.6 | 339.3 | . | . | . | . |
| a. Estimation is limited to the largest survival time if it is censored. | | | | | | | | |

| **Pairwise Comparisons** | | | | | | | | | | | | | |
| --- | --- | --- | --- | --- | --- | --- | --- | --- | --- | --- | --- | --- | --- |
|  | Marital status at diagnosis | Divorced | | Married (including common law) | | Separated | | Single (never married) | | Unmarried or Domestic Partner | | Widowed | |
|  |  | *Χ^2^* | Sig. | *Χ^2^* | Sig. | *Χ^2^* | Sig. | *Χ^2^* | Sig. | *Χ^2^* | Sig. | *Χ^2^* | Sig. |
| Log Rank (Mantel-Cox) | Divorced |  |  | 6.1 | .014 | .3 | .568 | 4.9 | .028 | .3 | .593 | 2.3 | .131 |
|  | Married (including common law) | 6.1 | .014 |  |  | .0 | .833 | .0 | .921 | .4 | .509 | 28.0 | .000 |
|  | Separated | .3 | .568 | .0 | .833 |  |  | .1 | .789 | .1 | .703 | 1.3 | .257 |
|  | Single (never married) | 4.9 | .028 | .0 | .921 | .1 | .789 |  |  | .4 | .504 | 17.2 | .000 |
|  | Unmarried or Domestic Partner | .3 | .593 | .4 | .509 | .1 | .703 | .4 | .504 |  |  | .0 | .842 |
|  | Widowed | 2.3 | .131 | 28.0 | .000 | 1.3 | .257 | 17.2 | .000 | .0 | .842 |  |  |

*Χ^2^ = Chi-Square, Sig. = Significance in p-value*

Supplementary table 5: Life Tables analysis for Cancer-Specific Survival

| Group/  Controls | Months | N Entering Interval | N  Withdrawing during Interval | N Exposed to Risk | N of Terminal Events | % Terminating | % Surviving | Cum. % Surviving at EI | SE of CP Surviving at EI | Pairwise Wilcoxon (Gehan) Sig. |
| --- | --- | --- | --- | --- | --- | --- | --- | --- | --- | --- |
| All Patients, MST = 240 m | 0 | 10556 | 3595 | 8758.5 | 1428 | 16.3% | 83.7% | 83.7% | 0.00 |  |
|  | 60 | 5533 | 1977 | 4544.5 | 576 | 12.7% | 87.3% | 73.1% | 0.01 |  |
|  | 120 | 2980 | 1353 | 2303.5 | 160 | 6.9% | 93.1% | 68.0% | 0.01 |  |
|  | 180 | 1467 | 666 | 1134.0 | 67 | 5.9% | 94.1% | 64.0% | 0.01 |  |
|  | 240 | 734 | 690 | 389.0 | 44 | 11.3% | 88.7% | 56.8% | 0.01 |  |
| A. Single, MST = 240 m | 0 | 1292 | 455 | 1064.5 | 162 | 15.2% | 84.8% | 84.8% | 0.01 | C, D |
|  | 60 | 675 | 242 | 554.0 | 67 | 12.1% | 87.9% | 74.5% | 0.02 |  |
|  | 120 | 366 | 165 | 283.5 | 19 | 6.7% | 93.3% | 69.5% | 0.02 |  |
|  | 180 | 182 | 73 | 145.5 | 8 | 5.5% | 94.5% | 65.7% | 0.02 |  |
|  | 240 | 101 | 91 | 55.5 | 10 | 18.0% | 82.0% | 53.9% | 0.04 |  |
| B. Married, MST = 240 m | 0 | 6517 | 2127 | 5453.5 | 862 | 15.8% | 84.2% | 84.2% | 0.00 | C, D |
|  | 60 | 3528 | 1209 | 2923.5 | 352 | 12.0% | 88.0% | 74.1% | 0.01 |  |
|  | 120 | 1967 | 877 | 1528.5 | 104 | 6.8% | 93.2% | 69.0% | 0.01 |  |
|  | 180 | 986 | 422 | 775.0 | 47 | 6.1% | 93.9% | 64.8% | 0.01 |  |
|  | 240 | 517 | 492 | 271.0 | 25 | 9.2% | 90.8% | 58.9% | 0.01 |  |
| C. Widowed, MST = 240 m | 0 | 1169 | 427 | 955.5 | 204 | 21.4% | 78.6% | 78.6% | 0.01 | A, B, D |
|  | 60 | 538 | 220 | 428.0 | 66 | 15.4% | 84.6% | 66.5% | 0.02 |  |
|  | 120 | 252 | 129 | 187.5 | 18 | 9.6% | 90.4% | 60.1% | 0.02 |  |
|  | 180 | 105 | 61 | 74.5 | 6 | 8.1% | 91.9% | 55.3% | 0.03 |  |
|  | 240 | 38 | 37 | 19.5 | 1 | 5.1% | 94.9% | 52.5% | 0.04 |  |
| D. Divorced, MST = 240 m | 0 | 719 | 248 | 595.0 | 102 | 17.1% | 82.9% | 82.9% | 0.02 | C |
|  | 60 | 369 | 138 | 300.0 | 50 | 16.7% | 83.3% | 69.0% | 0.02 |  |
|  | 120 | 181 | 89 | 136.5 | 12 | 8.8% | 91.2% | 63.0% | 0.03 |  |
|  | 180 | 80 | 39 | 60.5 | 3 | 5.0% | 95.0% | 59.9% | 0.03 |  |
|  | 240 | 38 | 32 | 22.0 | 6 | 27.3% | 72.7% | 43.5% | 0.06 |  |

N: Number, EI: End of Interval, Cum. %: Cumulative Proportion, %: Proportion, PD: Probability Density, SE: Standard Error, Sig.: Significance (p-value of the test)

Supplementary table 6: Details of Cox-Regression Model for overall survival (Model 1).

| **Omnibus Tests of Model Coefficients^a^** | | | | | | | | | |
| --- | --- | --- | --- | --- | --- | --- | --- | --- | --- |
| -2 Log Likelihood | Overall (score) | | | Change From Previous Step | | | Change From Previous Block | | |
|  | *Χ^2^* | df | Sig. | *Χ^2^* | df | Sig. | *Χ^2^* | df | Sig. |
| 65438.464 | 2386.597 | 13 | **.000** | 1825.767 | 13 | **.000** | 1825.767 | 13 | **.000** |
| a. Beginning Block Number 1. Method = Enter | | | | | | | | | |

|  | Sig. | HR | 95.0% CI for Exp(B) | |
| --- | --- | --- | --- | --- |
|  |  |  | Lower | Upper |
| Marital status at diagnosis | **.000** |  |  |  |
| Reference = Divorced |  |  |  |  |
| Marital status at diagnosis (Single) | **.004** | .806 | .694 | .935 |
| Marital status at diagnosis (Married) | **.000** | .727 | .645 | .820 |
| Marital status at diagnosis (Widowed) | .675 | .971 | .845 | 1.116 |
| Sex (Female) | **.000** | .790 | .740 | .843 |
| Age Group in 20s | **.000** |  |  |  |
| Reference = 80+ |  |  |  |  |
| Age Group (0-19) | **.000** | .072 | .041 | .126 |
| Age Group (20-39) | **.000** | .105 | .087 | .126 |
| Age Group (40-59) | **.000** | .205 | .184 | .228 |
| Age Group (60-79) | **.000** | .444 | .406 | .486 |
| Primary Site (Choroid) | .116 | .936 | .861 | 1.017 |
| SEER historic stage A | **.000** |  |  |  |
| Reference = Distant |  |  |  |  |
| SEER historic stage A (Local) | **.000** | .135 | .113 | .162 |
| SEER historic stage A (Regional) | **.000** | .237 | .193 | .290 |
| Rural Urban | .342 |  |  |  |
| Reference = Metropolitan |  |  |  |  |
| Rural Urban (Rural) | .184 | 1.153 | .934 | 1.424 |
| Rural Urban (Urban) | .484 | 1.033 | .944 | 1.130 |

HR: Hazard Ratio

Supplementary table 7: Details of Cox-Regression Model for Cancer-Specific survival (Model 1).

| **Omnibus Tests of Model Coefficients^a^** | | | | | | | | | |
| --- | --- | --- | --- | --- | --- | --- | --- | --- | --- |
| -2 Log Likelihood | Overall (score) | | | Change From Previous Step | | | Change From Previous Block | | |
|  | *Χ^2^* | df | Sig. | *Χ^2^* | df | Sig. | *Χ^2^* | df | Sig. |
| 31271.614 | 875.477 | 13 | .000 | 398.287 | 13 | .000 | 398.287 | 13 | .000 |
| a. Beginning Block Number 1. Method = Enter | | | | | | | | | |

|  | Sig. | HR | 95.0% CI for Exp(B) | |
| --- | --- | --- | --- | --- |
|  |  |  | Lower | Upper |
| Marital status at diagnosis | **.009** |  |  |  |
| Reference = Divorced |  |  |  |  |
| Marital status at diagnosis (Single) | .091 | .833 | .673 | 1.030 |
| Marital status at diagnosis (Married) | **.026** | .822 | .691 | .977 |
| Marital status at diagnosis (Widowed) | .875 | 1.017 | .823 | 1.256 |
| Sex (Female) | **.024** | .897 | .816 | .986 |
| Age Group in 20s | **.000** |  |  |  |
| Reference = 80+ |  |  |  |  |
| Age Group (0-19) | **.019** | .486 | .265 | .890 |
| Age Group (20-39) | **.000** | .508 | .399 | .646 |
| Age Group (40-59) | **.000** | .639 | .535 | .763 |
| Age Group (60-79) | **.011** | .807 | .684 | .951 |
| Primary Site (Choroid) | .237 | .928 | .819 | 1.051 |
| SEER historic stage A | **.000** |  |  |  |
| Reference = Distant |  |  |  |  |
| SEER historic stage A (Local) | **.000** | .093 | .075 | .116 |
| SEER historic stage A (Regional) | **.000** | .178 | .138 | .229 |
| Rural Urban | .348 |  |  |  |
| Reference = Metropolitan |  |  |  |  |
| Rural Urban (Rural) | .176 | 1.236 | .910 | 1.680 |
| Rural Urban (Urban) | .543 | 1.043 | .912 | 1.192 |

HR: Hazard Ratio

Supplementary table 8: Details of Cox-Regression Model for Overall and Cancer-Specific survival with considering tumor size (Model 2).

| **Summary** | **- Overall** |  |  |  | **Cancer-Specific Survival** | |  |  |
| --- | --- | --- | --- | --- | --- | --- | --- | --- |
|  |  | N | Percent |  |  |  | N | Percent |
| Cases available in analysis | Event^a^ | 348 | 3.3% |  | Cases available in analysis | Event^a^ | 182 | 1.7% |
|  | Censored | 1447 | 13.7% |  |  | Censored | 1595 | 15.1% |
|  | Total | 1795 | 17.0% |  |  | Total | 1777 | 16.8% |
| Cases dropped | Cases with missing values | 8762 | 83.0% |  | Cases dropped | Cases with missing values | 8762 | 83.0% |
|  | Cases with negative time | 0 | 0.0% |  |  | Cases with negative time | 0 | 0.0% |
|  | Censored cases before the earliest event in a stratum | 0 | 0.0% |  |  | Censored cases before the earliest event in a stratum | 18 | 0.2% |
|  | Total | 8762 | 83.0% |  |  | Total | 8780 | 83.2% |
| Total | | 10557 | 100.0% |  | Total | | 10557 | 100.0% |
| a. Dependent Variable: Survival months | | | |  | a. Dependent Variable: Survival months | | | |

| Omnibus Tests of Model Coefficients^a^ **for Overall Survival** | | | | | | | | | |
| --- | --- | --- | --- | --- | --- | --- | --- | --- | --- |
| -2 Log Likelihood | Overall (score) | | | Change From Previous Step | | | Change From Previous Block | | |
|  | Chi-square | df | Sig. | Chi-square | df | Sig. | Chi-square | df | Sig. |
| 4607.758 | 279.320 | 10 | 0.000 | 192.563 | 10 | 0.000 | 192.563 | 10 | 0.000 |
|  |  |  |  |  |  |  |  |  |  |
| Omnibus Tests of Model Coefficients^a^ for Cancer-Specific Survival | | | | | | | | | |
| -2 Log Likelihood | Overall (score) | | | Change From Previous Step | | | Change From Previous Block | | |
|  | Chi-square | df | Sig. | Chi-square | df | Sig. | Chi-square | df | Sig. |
| 2445.674 | 165.608 | 10 | 0.000 | 67.706 | 10 | 0.000 | 67.706 | 10 | 0.000 |

| **COX Model for Overall Survival** | | | | | |  | **COX Model for Cancer-Specific Survival** | | | | | |
| --- | --- | --- | --- | --- | --- | --- | --- | --- | --- | --- | --- | --- |
|  | Sig. | HR | 95.0% CI for Exp(B) | |  | |  | Sig. | HR | 95.0% CI for Exp(B) | |  |
|  |  |  | Lower | Upper |  | |  |  |  | Lower | Upper |  |
| Sex (Female) | **0.000** | 0.665 | 0.529 | 0.835 |  | | Sex (Female) | **0.015** | 0.679 | 0.497 | 0.927 |  |
| Age at diagnosis | **0.000** | 1.044 | 1.034 | 1.054 |  | | Age at diagnosis | 0.102 | 1.010 | 0.998 | 1.022 |  |
| Marital status at diagnosis | 0.066 |  |  |  |  | | Marital status at diagnosis | 0.307 |  |  |  |  |
| Marital status at diagnosis(Single) * | 0.312 | 1.268 | 0.800 | 2.009 |  | | Marital status at diagnosis(Single) * | 0.443 | 1.301 | 0.664 | 2.550 |  |
| Marital status at diagnosis(Married) * | 0.468 | 0.862 | 0.577 | 1.287 |  | | Marital status at diagnosis(Married) * | 0.610 | 1.164 | 0.650 | 2.082 |  |
| Marital status at diagnosis(Widowed) * | 0.715 | 1.095 | 0.672 | 1.784 |  | | Marital status at diagnosis(Widowed) * | 0.108 | 1.816 | 0.878 | 3.756 |  |
| Primary Site (Choroid) | **0.002** | 0.577 | 0.408 | 0.817 |  | | Primary Site (Choroid) | **0.006** | 0.525 | 0.331 | 0.833 |  |
| SEER stage (1973-2015) ^§^ | **0.000** |  |  |  |  | | SEER stage (1973-2015) | **0.000** |  |  |  |  |
| SEER stage (1973-2015)(Local) ^§^ | **0.000** | 0.073 | 0.043 | 0.125 |  | | SEER stage (1973-2015)(Local) ^§^ | **0.000** | 0.051 | 0.026 | 0.099 |  |
| SEER historic stage A (1973-2015)(Regional) ^§^ | **0.000** | 0.125 | 0.064 | 0.244 |  | | SEER historic stage A (1973-2015)(Regional) ^§^ | **0.000** | 0.082 | 0.034 | 0.197 |  |
| Basal Diameter | **0.001** | 1.001 | 1.001 | 1.002 |  | | Basal Diameter | **0.010** | 1.001 | 1.000 | 1.002 |  |
| Thickness (Depth) | **0.035** | 1.001 | 1.000 | 1.002 |  | | Thickness (Depth) | 0.232 | 1.001 | 0.999 | 1.002 |  |

* Compared to divorced

^§^ Compared to Metastatic

Supplementary table 9: Detailed Patients’ Characteristics.

MS Excel Sheet
